# Parameters of the Endocannabinoid System as Novel Biomarkers in Sepsis and Septic Shock

**DOI:** 10.3390/metabo7040055

**Published:** 2017-11-01

**Authors:** J. Daniel Lafreniere, Christian Lehmann

**Affiliations:** 1 Department of Pharmacology, Dalhousie University, Halifax, NS B3H 4R2, Canada; lafreniere@dal.ca; 2 Department of Anesthesia & Perioperative Medicine, Dalhousie University, Halifax, NS B3H 4R2, Canada

**Keywords:** sepsis, endocannabinoid system, septic shock, inflammation, immune system, biomarker, cannabinoid, immunomodulation

## Abstract

Sepsis represents a dysregulated immune response to infection, with a continuum of severity progressing to septic shock. This dysregulated response generally follows a pattern by which an initial hyperinflammatory phase is followed by a state of sepsis-associated immunosuppression. Major challenges in improving sepsis care include developing strategies to ensure early and accurate identification and diagnosis of the disease process, improving our ability to predict outcomes and stratify patients, and the need for novel sepsis-specific treatments such as immunomodulation. Biomarkers offer promise with all three of these challenges and are likely also to be the solution to determining a patient’s immune status; something that is critical in guiding effective and safe immunomodulatory therapy. Currently available biomarkers used in sepsis lack sensitivity and specificity, among other significant shortcomings. The endocannabinoid system (ECS) is an emerging topic of research with evidence suggesting a ubiquitous presence on both central and peripheral tissues, including an intrinsic link with immune function. This review will first discuss the state of sepsis biomarkers and lack of available treatments, followed by an introduction to the ECS and a discussion of its potential to provide novel biomarkers and treatments.

## 1. Introduction

Sepsis represents a dysregulated immune response to infection, with a continuum of severity progressing to the most severe and refractory state of septic shock. There are several key challenges in attempting to improve sepsis care. These include strategies to ensure early and accurate identification and diagnosis of the disease process, strategies to predict outcomes and stratify patients, as well as developing novel treatments and treatment strategies, of which there are none on the market to date. Biomarkers offer promise with all three of these challenges. Currently available biomarkers used in sepsis suffer from low specificity and or sensitivity, among other shortcomings. These include the three most used biomarkers for sepsis in clinical practice: procalcitonin (PCT), C-reactive protein (CRP) and lactate. In particular, determining an accurate and “real-time” picture of a patient’s immune status is critical in order to guide effective immunomodulatory therapy, while minimizing the potential complications of these interventions. The endocannabinoid system (ECS) is a rapidly emerging topic of research, with growing evidence suggesting an intrinsic link with immune function, particularly through activation of the cannabinoid 2 Receptor (CB2R). There are several parameters of the ECS that may serve as novel biomarkers in sepsis: levels of the endocannabinoids (ECs) themselves, levels of the enzymes responsible for EC synthesis and EC degradation, and expression levels of the cannabinoid receptors.

### 1.1. Sepsis

The Third International Consensus Definitions for Sepsis and Septic Shock (Sepsis-3) defines sepsis as life-threatening organ dysfunction caused by a dysregulated host response to infection [1]. Usually, a dysregulated immune response manifests as a hyperinflammatory immune state that is frequently followed by a state of sepsis-associated immunosuppression. Sepsis is centrally associated with an evolving and complex immunopathology, progression of which relies heavily on each patient’s immune status. In particular, the scale of an initial hyperinflammatory phase response, if present, has been shown to correlate with pathogen-specific factors (such as virulence or size of the inoculum), and host-specific factors such as age, or comorbidities including/in addition to the patient being immunocompromised before developing sepsis [2]. In some patients, such as those with dysfunctional immune systems (chronic disease, pharmacologically-induced immunosuppression etc.), it is possible that no initial hyperinflammatory state of sepsis will manifest, resulting in the patient progressing directly to a state of sepsis-associated immunosuppression. Expanding on this, aging of the immune system—known as ‘immunosenescence’ [3] has been linked to higher rates of mortality in the >65 year old population of sepsis patients [2]. The severity of sepsis can progress along a continuum to septic shock, characterized by profound circulatory, cellular, and metabolic abnormalities and associated with an increased risk of mortality than that in sepsis alone [1]. 

The burden of sepsis is pronounced, with high mortality rates and an increasing incidence [4]. Sepsis is seen ubiquitously through healthcare settings, however particularly in the adult intensive-care unit (ICU) where sepsis-related organ failure accounts for approximately 50% of deaths [5]. Similarly, the burden is particularly high in the surgical ICU, where sepsis and septic shock constitute the leading cause of mortality internationally [6]. Mortality is especially common during the state of sepsis-associated immunosuppression [2], possibly a result of the resurgence of the initial sepsis-inciting infection and/or development of nosocomial infection(s); however, mortality also occurs in the hyperinflammatory state. Interestingly, little is known relating to the cause of death in sepsis patients, representing another gap in our understanding of the pathophysiology. We do, however, know from autopsy studies that most patients admitted to the ICU for sepsis treatment have unresolved septic foci [7], and that the etiology of these foci can be either nosocomial or stem from the original infection [2]. 

### 1.2. State of Sepsis-Specific Treatments & Considerations with Immunomodulators

The mainstay of sepsis treatment centers on early and empiric initiation of broad-spectrum antibiotics, aggressive fluid resuscitation, and supportive care as needed for organ system dysfunction (i.e., intubation & ventilation for respiratory dysfunction such as ALI/ARDS, dialysis for renal failure, or vasopressors for refractory hypotension etc.). The introduction of novel sepsis treatments and treatment strategies does not mirror the prevalence and burden of the disease, with no effective treatments currently available on the market. Treatments based on a range of approaches have resulted in failure, such as attempting the use of anti-inflammatory cytokines aimed at dampening cytokine cascades during the initial hyperinflammatory phase, or the use of agents aimed at normalizing dysregulated coagulation cascades [8]. The FDA approval of drotrecogin alfa (activated Protein C) generated interest after benefits were observed following treatment of sepsis patients [9]; however this agent was withdrawn from the market in 2011 after a follow-up study failed to identify any benefit [10]. Further, a trial examining the efficacy of the blockade of Interleukin-1 (IL-1) using Anakinra (a recombinant human IL-1 receptor antagonist) was terminated early as the group failed to demonstrate reduction in mortality after a 72-h continuous infusion [11].

The search for sepsis-specific therapies is overarched by the pathophysiological complexity of sepsis as well as the high patient-to-patient variability of disease course. It is possible that simplistic treatment strategies such as blockade of a specific cytokine fail as they do not take into account the complexity and resilience of the sepsis disease process. This being said, immunomodulatory therapy holds great promise in sepsis due to the central role of immunopathology in sepsis. The use of biomarkers hold potential for improving our ability to accurately and quickly diagnose sepsis, to predict disease severity (such as progression to septic shock), as well as for guiding treatment—such as assessing the state of the patient’s immune system in order to guide immunomodulatory therapy. A key consideration when developing and implementing immunomodulatory therapies in sepsis is a “real time” picture of the patient’s immune status. We know that immunosuppression or ‘immunoparalysis’ (when the patient fails to mount/sustain an immune response in the presence of unresolved infectious foci) is not the time to administer an immunosuppressive treatment, as this would make it more difficult for the patient to clear the sepsis-inciting infection, and also increase their susceptibility to nosocomial infections [12]. Similarly an immune-bolstering treatment could theoretically lead to a further exaggerated hyperinflammatory response, if administered during this phase.

### 1.3. Biomarkers in Sepsis

#### 1.3.1. Overview

There are no sepsis-specific biomarkers currently available on the market. There are also no biomarker(s)/biomarker panels that offer a comprehensive assessment of the immune status in sepsis patients. Current biomarkers used in clinical practice assess systemic inflammation/infection in a non-specific way, and lactate functions to assess cellular metabolic dysfunction, which must be interpreted in the context of other clinical information. A 2010 review by Pierrakos & Vincent reported that there were over 170 different potential sepsis biomarkers reported at that time [13].

#### 1.3.2. As Diagnostic Markers

Currently, the diagnosis of sepsis or septic shock is made based on clinical examination, a patient history, and laboratory studies that are collected upon initial identification of suspected sepsis. These lab investigations include complete blood count with auto-differential, chemistries and liver function tests, coagulation studies including D-dimer, an arterial blood gas panel, aerobic and anaerobic blood cultures, urinalysis, microbiologic cultures (e.g., from area surrounding a suspected catheter), aerobic and anaerobic cultures obtained from multiple sites, and a serum lactate level [14]. In addition to these tests, some centers run C-reactive protein (CRP) and/or procalcitonin (PCT) [15].

Once there is suspicion that a patient has sepsis and initial investigations are ordered, there are still various roadblocks in confirming the diagnosis and obtaining accurate information that can be used to guide specific treatments. For example, the extended study of prevalence of infection in intensive care (‘EPIC II’) trial performed in 2007 found that negative cultures were reported in 30–40% of ICU patients with severe sepsis [6]. This high rate of culture-negative infection has the potential to be misinterpreted as the absence of active infection. Further, without confirmation of the infective agent, antimicrobial regimens cannot be tailored.

#### 1.3.3. Goals in Biomarker Selection & Multi-Biomarker Panels

Ideal biomarkers used in the diagnosis of sepsis would be highly sensitive and specific. Standardization of the biomarkers is important in the context of multicenter investigations and in allowing the markers to perform reliably across diverse patient populations. These markers may additionally offer insight into the infectious etiology, independent of the culture-positivity of the pathogen. A biomarker that offers prognostic insight could be a useful guide in stratifying patient populations and tailoring treatment regimens. With respect to immunomodulatory therapy, markers that report real-time characterization of immune response and overall immune activation status are required to accurately and safely guide treatment. As an aside, these biomarkers may also function to report efficacy of targeted sepsis treatments and/or offer valuable insight into the pathophysiology of sepsis.

It is likely that a single biomarker approach to sepsis is impractical due to the complexity of the pathophysiology and patient-to-patient variability. Multi-biomarker panel approaches to sepsis have been proposed and may overcome these challenges [15]. Each marker in a panel could be selected based on specific strengths, where another marker could compensate for its shortcomings. Further, the development of algorithms for interpretation of these panels could in theory improve sensitivity and specificity by accounting for patient-specific factors in diagnosis, such as presence of immunosuppression, the patient’s age, and could additionally account for pathogen-specific factors.

#### 1.3.4. Key Markers in Clinical Practice

##### Procalcitonin (PCT) & C-Reactive Protein (CRP)

Procalcitonin (PCT) and C-reactive protein (CRP) are the most frequently used biomarkers in the diagnosis and management of sepsis [16]. PCT and CRP are both proteins produced during a normal physiologic inflammatory response, or during the response to infection. CRP was discovered when it was noticed in increased levels in patients with pneumococcal pneumonia [17,18] and is classified an ‘acute phase reactant’—a term describing serum proteins that increase in situations of tissue injury or inflammation [19,20]. The role of CRP itself in acute inflammatory events is unclear, though it may be directly involved in pathogen or damaged host cell clearance by acting to opsonize them for phagocytosis and/or through direct activation of the classical complement pathway [15]. PCT, on the other hand, is the precursor to the mature hormone calcitonin, produced by many tissues including those not at the site of inflammation [21]. PCT was first noted as a potentially useful marker when it was found to be elevated in patients with invasive bacterial infections [22]. In fact, PCT has itself been shown to be involved in the process of a systemic response leading to sepsis [23].

Despite their popularity, the consensus is that both PCT and CRP lack sensitivity and particularly lack specificity for sepsis [24], and further suffer from limitations such as PCT’s variable levels in early stages of sepsis—limiting its value to use in later stages of sepsis [25,26]. One possible advantage of PCT over CRP is that it appears to have specificity for bacterial infection, and therefore may play a role in recognition of a bacterial infection prior to culture results becoming available [27]. Garnacho–Montero et al. explain that effectively characterizing infective processes compared to inflammatory processes is an important goal in the ICU, as an inflammatory response is seen to some extent in most critically ill patients [16]. CRP is a well-established non-specific marker of inflammation, with sensitivity for bacterial infections usually listed around 80% [27]. As a marker of infection the sensitivity of CRP has (at >8.7 mg/dL) been reported at 93.4% [28]. In particular CRP has a very high specificity for identifying sepsis in the neonatal setting [29]. Both CRP assays and recently PCT immunoassays are becoming more accessible, and despite debate there is no consensus on which test is superior [15]. Pierrakos and Vincent highlight that there are not many biomarkers with good diagnostic value being examined, and that our currently available markers instead hold more value in prognosis [13]. 

##### Lactate

Serum lactate is routinely used as a biomarker in sepsis diagnosis and management [14]. Evidence suggests not only a value in the use of lactate for sepsis diagnosis but also that it functions well as a prognostic marker for sepsis-related mortality [30]. Lactate is a well-established marker for organ dysfunction and was recently incorporated into the diagnostic criteria guidelines for septic shock, as part of the Sepsis-3 definitions [1]. Specifically, Sepsis-3 defines septic shock as the need to use vasopressors to maintain a mean arterial pressure above 65 mmHg concurrent with a serum lactate of >2 mmol/L, in the absence of hypovolemia. These guidelines follow knowledge that hyperlactatemia in those sepsis patients resistant to fluid resuscitation are at a particularly high risk of mortality [31]. In fact, once a patient meets the Sepsis-3 criteria of septic shock, their predicted mortality rises to >40% [1]. It is important to note that despite widespread belief that hyperlactatemia results from hypoxic tissues shifting to anaerobic glycolysis [31], new theories suggest that this is instead resulting from dysregulated metabolic processes—specifically from an increased adrenergic tone that leads to an excess of pyruvate from increased aerobic glycolysis [32,33]. Sepsis-induced liver dysfunction may play a role in elevated lactate levels in sepsis, due to impaired hepatic lactate clearance [34]. 

##### Assessing Immune Status & Function in Sepsis: A Guide for Immunomodulatory Therapy

Establishing reliable biomarkers that offer a “real-time” picture of immune status is required to accurately guide immunomodulatory therapy in sepsis. Inappropriate timing of therapy may lead to exacerbation of the patient’s immune dysregulation—through bolstering the immune response of a hyperinflammatory state patient. Similarly, the use of anti-inflammatory agents may potentiate the sepsis-associated immunosuppression that characterizes the second phase of sepsis [6]. Assessing immune status would likely rely on a panel of biomarkers and at least in part utilize flow cytometry. Flow cytometry rapidly generates a range of specific information regarding immune cells, including quantification, functional and activation status analysis, as well as an analysis of immune cell populations, all of which can be generated from peripheral blood samples.

In addition to early biomarkers of severe inflammation (PCT and CRP), it is important to acknowledge biomarkers of later stages of sepsis indicative of progression to a state of sepsis-associated immunosuppression. These biomarkers would be essential to guide safe and effective immune-bolstering treatments. Decreases in cell surface expression of the major histocompatibility complex—class II (MHC-II) receptor, monocyte human leukocyte antigen-D related (mHLA-DR), is considered the best marker of sepsis-associated immunosuppression available to date [35]. Monocytes are immune cells known to play key roles in both innate and adaptive immune responses. Illustrating the link between monocyte function and immune dysregulation in sepsis are studies demonstrating changes in parameters of monocyte function, in addition to diminished expression of mHLA-DR. During a state of sepsis-associated immunosuppression, these parameters include reductions in phagocytic action and altered cytokine expression—with a shift towards production of anti-inflammatory cytokines [36]. Quantification of mHLA-DR can be performed through flow cytometry, which offers a fast turnaround time.

## 2. The Endocannabinoid System (ECS)

**Figure 1:**
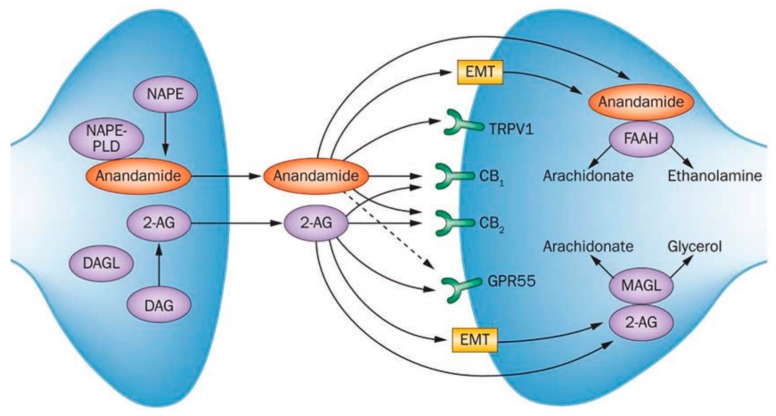
Summary of the endocannabinoid system; highlights key endocannabinoids, cannabinoid receptors, and synthesis/degradative enzymes. NAPE: *N*-acyl-phosphatidylethanolamine; NAPE-PLD: *N*-acyl-phosphatidylethanolamine-specific phospholipase D; 2-AG: 2-arachidonoylglycerol; DAG: diacylglycerol; DAGL: diacylglycerol lipase; EMT: endocannabinoid membrane transporter; TRPV1: transient receptor potential cation channel subfamily V member 1; CB1: cannabinoid receptor 1; CB2: cannabinoid receptor 2; GPR55: G protein-coupled receptor 55; FAAH: fatty acid amide hydrolase; MAGL: monoacylglycerol lipase. From: Schicho, R. & Storr, M. Patients with IBD find symptom relief in the cannabis field. *Nat. Rev. Gastroenterol. Hepatol.* 2013. doi:10.1038/nrgastro.2013.245 [51].

The ECS is a ubiquitous and complex system with a rapidly growing body of evidence suggesting an intricate relation with a range of physiologic and disease processes. Cannabinoids have been used for medical purposes for over 6000 years, and we are now aware of over 100 plant-derived cannabinoids (known as phytocannabinoids), including the psychoactive compound tetrahydrocannabinol (THC) [37]. The discovery of endogenous bioactive lipids (endocannabinoids—ECs) that acted on the receptors responsible for THC’s actions led to the notion of a human cannabinoid system. The two key ECs are anandamide (AEA) and 2-arachidonylglycerol (2-AG), and these along with other ECs, the cannabinoid receptors, and various enzymes responsible for EC synthesis and degradation compose the ECS (Figure 1). Cannabinoid receptors include the cannabinoid type 1 receptor (CB1R)—primarily localized to the brain and spinal cord, and which is the receptor responsible for the psychotropic effects of THC, and secondly the cannabinoid type 2 receptor (CB2R)—primarily restricted to immune cells in the periphery. These two receptors share 44% amino acid homology [38]. 

CB1R and CB2R are activated endogenously by both AEA and 2-AG, and exogenously by THC and other cannabinoids [39,40]. Both CB1R and CB2R are G-protein coupled receptors (GPCRs), and in the CNS the activation of CB1R is associated with Gαi protein coupled downstream signaling pathways, including adenylyl cyclase (AC), cyclic adenosine monophosphate (cAMP), and mitogen activated protein kinase (MAPK) [40]. A retrograde signaling mechanism has been hypothesized and leads to the inhibition of presynaptic voltage-gated Ca^2+^ channels, which in turn leads to a decrease in the fusion of neurotransmitter vesicles and thus decreased synaptic neurotransmitter release [41]. CB2R also signals through receptor coupling to Gαi proteins and results in the inhibition of AC, leading to a decrease in levels of cAMP [42]. Within myeloid cells, cAMP has a key regulatory role in p38 and nuclear factor-kappa B (NF-κB). Decreased levels of cAMP (as seen with CB2R activation) appear to enhance neutrophil activation [43] and enhance bacterial clearance through phagocytosis [44]. In contrast, elevations in cAMP levels (as seen with CB2R blockade) appear to reduce the phagocytic and oxidative burst action of neutrophils, impairing their ability to clear bacteria [43]. The CB2R also functions in several second messenger systems, including MAPK, nitric oxide synthase (NOS), Ca^2+^ channel-related signaling, and phospholipase C (PLC), all which function as signaling pathways in immune cells [45].

Generation of ECs occurs through enzymatic action on membrane lipids in an on-demand process [41], and their duration of action is dictated by enzymatic degradation. Formation of 2-AG relies on the production of 1,2-diacylglycerol (DAG) through enzymatic action, followed by conversion of DAG to 2-AG by DAG lipase [46,47]. Formation of AEA occurs through phospholipase D’s enzymatic action on N-arachidonyl phosphatidylenthanolamine (NAPE) [48]. The key enzymes involved in EC degradation are fatty acid amide hydrolase (FAAH), which degrades AEA; and monoacylglycerol lipase (MAGL), which degrades 2-AG [49,50].

## 3. Immune System & the ECS in Sepsis

There is a strong link between the ECS and immune function, with emerging evidence contributing to our understanding [52]. CB2R is the cannabinoid receptor most discussed in relation to inflammation and presence on immune cells. This presence is ubiquitous and the following lists immune cells in descending order of CB2R expression levels: natural killer cells, monocytes, polymorphonuclear leukocytes, CD4+ and CD8+ lymphocytes [53]. In relation to sepsis, it is important to consider that CB2R modulation may lead to different effects based on both the timing of the intervention and on the particular disease course in that individual—with particular note to the severity of disease [54]. Evidence suggests that activation of CB2R leads to a reduction in the recruitment of neutrophils and macrophages (early responders in innate immune activation), as well as a reduced generation of pro-inflammatory cytokines, while at the same time promoting bacterial clearance through increasing phagocytosis [44,55]. 

We also know that various models of injury trigger increased expression levels of CB2R and of ECs in the circulation, which may indicate a protective role of the ECS in response to endogenous inflammation [56,57]. Studies have indicated that the main ECs, AEA and 2-AG, lead to decreased pro-inflammatory cytokine release by macrophages and microglial cells following an aseptic gram-negative (lipopolysaccharide (LPS)-induced) model of sepsis, as well as that 2-AG inhibited the release of the pro-inflammatory cytokine IL-2 from murine splenocytes [58,59,60]. Regarding modulation of cannabinoid-related enzymes, the inhibition of FAAH (via the selective FAAH inhibitor URB597) was shown to result in a reduced expression of inflammatory mediators in rat cortical microglia, using an LPS-induced sepsis model [61] and similarly decreased levels of TNFα and IL-1β in an LPS-induced pain model in rats [62].

Potential for ECS-based sepsis immunomodulatory therapy likely involves modulation through the CB2R, its EC ligands, or the enzymes responsible for their formation and degradation. Sepsis biomarkers using the ECS may offer a unique set of information relating to immune status, and/or may be useful in guiding and assessing effects of ECS-targeted immunomodulation.

### 3.1. The ECS as a Tool in Sepsis

#### 3.1.1. Biomarkers of the ECS 

##### Endocannabinoids

In a clinical setting, assessing levels of ECs would most practically be done using peripherally obtained whole blood. The highly lipophilic nature of ECs and other cannabinoids mean that lipid extracts would be isolated from whole blood plasma or serum prior to running assays for EC quantification. Typical laboratory methods for the measurement of ECs would use these lipid extracts and perform isotope-dilution liquid chromatography-mass spectrometry [63]. Several methods for EC extraction from serum and plasma have been published [64]. We are aware of some considerations when assessing levels of ECs. For example, the measurement of 2-AG has been shown to be more reliable when the sample is isolated from the serum [65]. Hillard also highlights evidence suggesting that AEA concentrations are higher in serum than in plasma [66]. Further, evidence indicates that a time-dependent concentration change of AEA after delay between blood draw and cell separation [67] may result from the red blood cell’s ability to release and inactivate AEA—leading to the reasonable assumption that time between the collection and testing should be minimized [63]. There is also evidence to suggest that the levels of ECs vary significantly in healthy individuals over the course of a day, and that these are influenced by factors such as exercise and food intake [63]. The normal serum reference ranges for AEA are reported to be 1–5 nM and for 2-AG are reported to be 10–500 nM [65].

##### Enzymes

Assessing levels of the enzymes involved in EC synthesis and degradation may offer benefit over directly assessing EC levels. Because ECs are synthesized in an on-demand fashion via enzymatic action on membrane lipids, an increase in EC synthesis enzymes, such as DAG lipase—for 2-AG or phospholipase D—for AEA, would most likely precede increases in the ECs themselves. This could mean that EC enzyme levels might offer a more expedient indication of ECS activation than those offered by quantifying levels of ECs. Studies indicate that enzymes associated with EC synthesis or degradation can be assessed from whole blood samples using enzyme activity assays [68,69].

##### Expression of Cannabinoid Receptors

The levels of the cannabinoid receptors, CB1R & CB2R, could also be assessed as markers of ECS activation. Techniques used for quantification of receptor expression include immunofluorescence assays—involving antibodies targeted against the specific receptor, or the use of a quantitative and specific reverse transcription PCR (RT-PCR) technique—involving oligonucleotide probes specific to conserved regions of the respective receptor’s mRNA [53]. Regarding the practicality of these assays in a clinical scenario, CB2R would be quantifiable through isolation of white blood cells (WBCs) from venous whole blood. As a marker of immune status this WBC isolation would be practical and time-efficacious, pending the strategy used for quantification of receptor expression was optimized for an acceptable turn-around time and cost. 

#### 3.1.2. Theoretical Mechanisms of ECS-Based Immunomodulatory Therapy in Sepsis

Theoretically, the ECS could function as a target of immunomodulatory therapy in either phase of sepsis. In the initial hyperinflammatory phase, potential strategies may include direct activation of CB2R using selective agonists, or inhibitors of the EC-degrading enzymes—which would lead to increased levels of ECs. Implementing these strategies could have potent and fast-onset systemic anti-inflammatory action. This anti-inflammatory action may be effective in: dampening the direct cytotoxic damage from a hyperinflammatory response, normalizing immune function and enhancing clearance of the pathogen, as well as in preventing the excessive production of an endogenous anti-inflammatory response—which could contribute to the prevention of the development of a state of sepsis-associated immunosuppression.

A logical goal during the state of sepsis-associated immunosuppression is to stimulate the immune system and facilitate a return of function. This would help promote clearance of the original infection and prevent establishment of new nosocomial infection(s). More evidence is available that would suggest the use of ECS modulation as an anti-inflammatory to be used during the hyperinflammatory phase of sepsis [70]. Should an ECS-based approach be applied to target the state of sepsis-associated immunosuppression, it would likely involve the blockade of CB2R or strategies to decrease production and/or increase the degradation of ECs. It is important to recall that ECS-based immunomodulation in sepsis, as the case with all immunomodulatory strategies, would need to be carefully timed and initiated based on an accurate picture of the patient’s immune status. 

Regarding cannabinoids as medical therapeutics, it is underappreciated that, to be non-psychoactive, a cannabinoid need only lack the ability to activate central CB1Rs. Cannabinoid compounds can be designed to have higher affinity for particular receptors and could also be molecularly modified to remove their ability to cross the blood-brain-barrier. Given this, peripherally restricted CB1R modulation remains a non-psychoactive option. Similarly, CB2R modulation is a non-psychoactive option. It is important when considering psychoactive effects of cannabinoids to appreciate that many sepsis and other ICU patients are under some level of sedation.

## 4. Conclusions

Biomarkers may be the most effective novel area of discovery in the development of techniques for the diagnosis and treatment of sepsis. A particular goal is developing biomarkers or multi-biomarker panels with high sensitivity and specificity for sepsis, and which function reliably across diverse patient populations and infectious etiologies. The ECS represents an increasingly researched and appreciated system with a ubiquitous presence, including on a range of immune cells. Using parameters of the ECS as biomarkers in sepsis holds potential, with research into this topic potentially offering insight into the immune pathophysiology of sepsis. A better understanding of the immunopathology of sepsis would be invaluable in the discovery of novel immunomodulatory therapies.
